# Southern Medical and Surgical Journal

**Published:** 1845-02

**Authors:** 


### SOUTHERN MEDICAL AND SURGICAL JOURNAL.

We have received the first number of a *new series* of the Southern Medical and Surgical Journal, published at Augusta, Georgia, and edited by Paul F. Eve, M. D., and J. P. Garvin, M. D. A Journal with this title was commenced in the year 1836, under the editorial management of the late Professor Antony, but, on the occurrence of his death, was discontinued. After the failure of several attempts, it is now revived, and, it is believed by the Editors, under arrangements which will render its future continuance certain. It is to be issued monthly, at three dollars per annum. The number before us contains forty-eight octavo pages of matter, original and selected, on various subjects embraced in Medical Science. The Editors are well known as able teachers and writers, and long conversant with the diseases of the South, and we hope for the sake of the profession in all parts of the country, but more especially in the interesting region where they reside, that their present attempt will be successful.

